# Recombinant inbred systems can advance research in behavioral ecology

**DOI:** 10.3389/fgene.2012.00198

**Published:** 2012-10-04

**Authors:** Beatrice Gini, Reinmar Hager

**Affiliations:** Computational and Evolutionary Biology, Faculty of Life Sciences, University of ManchesterManchester, UK

**Keywords:** QTL, behavioral ecology, BXD, systems genetics, recombinant inbred

## Abstract

Recombinant inbred (RI) systems such as the BXD mouse family represent a population with defined genetic architecture and variation that approximates those of natural populations. With the development of novel RI lines and sophisticated methods that conjointly analyze phenotype, gene sequence, and expression data, RI systems such as BXD are a timely and powerful tool to advance the field of behavioral ecology. The latter traditionally focused on functional questions such as the adaptive value of behavior but largely ignored underlying genetics and mechanisms. In this perspective, we argue that using RI systems to address questions in behavioral ecology and evolutionary biology has great potential to advance research in these fields. We outline key questions and how they can be tackled using RI systems and BXD in particular. The unique opportunity to analyze genetic and phenotypic data from studies conducted in different laboratories and at different times is a key benefit of RI systems and may lead the way to a better understanding of how adaptive phenotypes arise from genetic and environmental factors.

## Introduction

The field of behavioral ecology seeks to understand causes and consequences of variation in complex behavioral phenotypes with a focus on ecological and social conditions to which behavior may be adapted (Krebs and Davies, 1978; Davies et al., 2012; Hager and Gini, 2012). To this end, studies have long relied on what Grafen (1984) termed the “phenotypic gambit;” the assumption that the phenotypic traits in question, be they life history strategies or behavioral patterns, are reflective of their underlying genetics, the details of which, however, are largely irrelevant to the models and predictions in question (Owens, 2006; Smiseth et al., 2008). Indeed, if the sole objective is to understand the adaptive value of behavior and test model predictions empirically it has been demonstrated that one can perfectly well investigate the fitness costs and benefits of behavior without reference to genetics (Kacelnik, 1984; Dreiss et al., 2010). However, some cases violate the assumptions of the gambit, to the point where the gambit's predictions are wrong (Hadfield et al., 2007; Gratten et al., 2008). Moreover, recent advances in genetics, epigenetics and bioinformatics have shown that the evolution of many traits can only be modeled accurately when we take into account their genetic architecture.

## Advancing behavioral ecology using RI systems

With the increased development of genetics and genomics tools, research in behavioral ecology in particular and evolutionary biology in general can be advanced by going beyond Grafen's gambit and adopting some of the technologies developed in other disciplines (e.g., Hager et al., 2012). While this argument is far from new and, for example, quantitative genetic approaches have been used for over 20 years to address questions in behavioral ecology (e.g., Boake, 1994; Boake et al., 2002), with the development of advanced recombinant inbred (RI) systems and genome sequencing the range of questions can be expanded, and we are able to investigate in much greater detail how adaptive effects arise. Thus, in addition to the traditional focus on functional (adaptive) questions, we can now better study the evolution of behavior, proximate mechanisms, and ontogeny as outlined in Tinbergen's (1963) four questions: (1) causation: what immediate causes lead the organism to perform the behavior (proximate causes)? (2) survival value: what is the adaptive advantage associated with a behavior (functional causes)? (3) evolution: how has the behavior evolved in the species' phylogeny? and (4) ontogeny: how does a behavior arise during the organism's development?

Among the key questions are how does plasticity enable some individuals to better respond to environmental variation, and how, at a genetic level, is plasticity achieved? Which traits are plastic and which not, and why? What types of genetic variants are most often involved in evolutionary change and adaptation? How do complex behavioral phenotypes develop during ontogeny and how much of their variation is due to genetic versus environmental variation? To what extent do genes help shape the social environment in which individuals live, and what are the consequences of this for behavioral traits and their adaptation to this environment? Are complex behavioral phenotypes constrained in their response to selection (thus producing a seemingly suboptimal phenotype) and are there genetic constraints? Further, with novel genomics and bioinformatics tools such as network analyses, one can now start to establish the pathways involved from sequence variation to intermediate phenotypes (e.g., physiological traits) through to complex phenotypes such as behavior. We give brief examples of how to tackle some of the above questions in Box 1 with a worked example in Figure 2.

Box 1 **Behavioral ecology questions that can be addressed in RI systems.**

- **Genetic basis of trait variation.** What genetic variants are associated with phenotypic variation? This requires the use of a genome-scan (Figures 2A and 2B). Trait values obtained in an experiment are entered into GeneNetwork, which correlates them to variation in the genotype between BXD lines. The genomic regions with the highest correlation are identified visually as peaks in the LRS score (i.e., the location of a QTL, Haley and Knott, 1992). Heritability can be assessed in a simple ANOVA using *line* as the independent variable and the trait of interest as the dependent variable, then dividing the between line variance by the total variance. Once candidate genes are identified by selecting biologically relevant candidates listed under the peak of the QTL with the highest LRS score, their sequence can be examined using the interval analyst tool, which displays substitutions, insertion and deletions. The next step is to find out whether the mutation affects the phenotype directly, or whether it acts via the expression of a second gene. Mutations in genes that code for extra-nuclear proteins can be distinguished by those in regulatory genes by using the links to other databases such as the NCBI. These provide a short description of the gene status (e.g., “protein coding”) as well as links to the literature describing gene function in more detail. Large expression datasets are available and can be correlated to gene sequences.
- **Phenotypic plasticity.** How is plasticity achieved at a genetic level? Measuring behavior or other life history traits in multiple lines across a gradient of environmental conditions allows genome-scans (Figure 2A) for these traits that can identify genetic variants modifying traits in some but not other environments. Correlation (Figure 2D) of the measured traits with e.g., physiological traits and gene expression levels may reveal information about why some traits are more plastic than others and with which gene expression levels the phenotypes correlate. By phenotyping animals during development one can establish which genes play a role at what stage in development; varying environmental conditions for each of the lines can identify environmental dependency of genetic variants in their effects on phenotypes.
- **Constraints.** Are complex behavioral phenotypes constrained in their response to selection and why? Genetic constraints can be explored by assessing specific epistatic interactions (see below) and determining which traits are affected by interacting genes using correlation of gene expression levels and phenotypes. Because phenotypes other than those measured in a specific experiment are available for BXD, as are gene expression data for major brain parts, correlational analyses in RI panels are much more comprehensive than could be in any single experiment.
- **Epistasis.** A specific pairwise mapping tool calculates and displays correlations between gene pairs and phenotype and comparison between main effects of genes and interaction effects (Figure 2C).

Traditionally, research on the genetic and environmental basis of behavioral traits has used crosses of model organisms that are divergent in the expression of the trait of interest (e.g., low and high aggressiveness; Brodkin et al., 2002), phenotyping of knock-out mutants for one or two candidate genes or breeding designs that allow the estimation of heritability, i.e., the proportion of phenotypic variation that can be explained by genetic variation. In wild populations with complex pedigrees, the animal model has been used to partition variance components (e.g., Kruuk, 2004). All of these approaches rely on data of a population that is genetically unique in its variation among individuals and in its architecture. Thus, any new experimental population needs to be geno- and phenotyped, and results of different studies, even using the same founders, cannot be pooled easily due to differences in genotypes in the population. RI systems circumvent this problem as the genetic variation is fixed (hence inbred) for a given population and animals bred from this population can be used in future research knowing that the genetic variation in the entire population remains constant and defined.

In this perspective we outline the advantages of using RI populations for studies in behavioral ecology integrating information about multiple genes, phenotypes, and environmental factors. Thus, our aim is to bring together the advances made in genetics and the conceptual framework of behavioral ecology. We focus on a mammalian RI system, the C57Bl6/J crossed with DBA (BXD) mouse population, because it is by far the largest model system both in terms of genetic and analytical resources. Most importantly, however, is that the BXD system is the most widely applicable for research in mammalian behavioral ecology and can be used by behavioral ecologists and animal behaviorists without genetic background. Of course, other systems such as recombinant congenic or chromosome substitution lines in mice may be more suitable for particular questions and insect RI lines have also been extensively used for some time (e.g., *Drosophila*; Gleason et al., 2002). Nevertheless, BXD enables the rapid integration and analysis of experimentally obtained phenotype data with genetic and gene expression data without the need to generate the latter in a specific experiment.

## The BXD recombinant inbred model system

**Figure 1:**
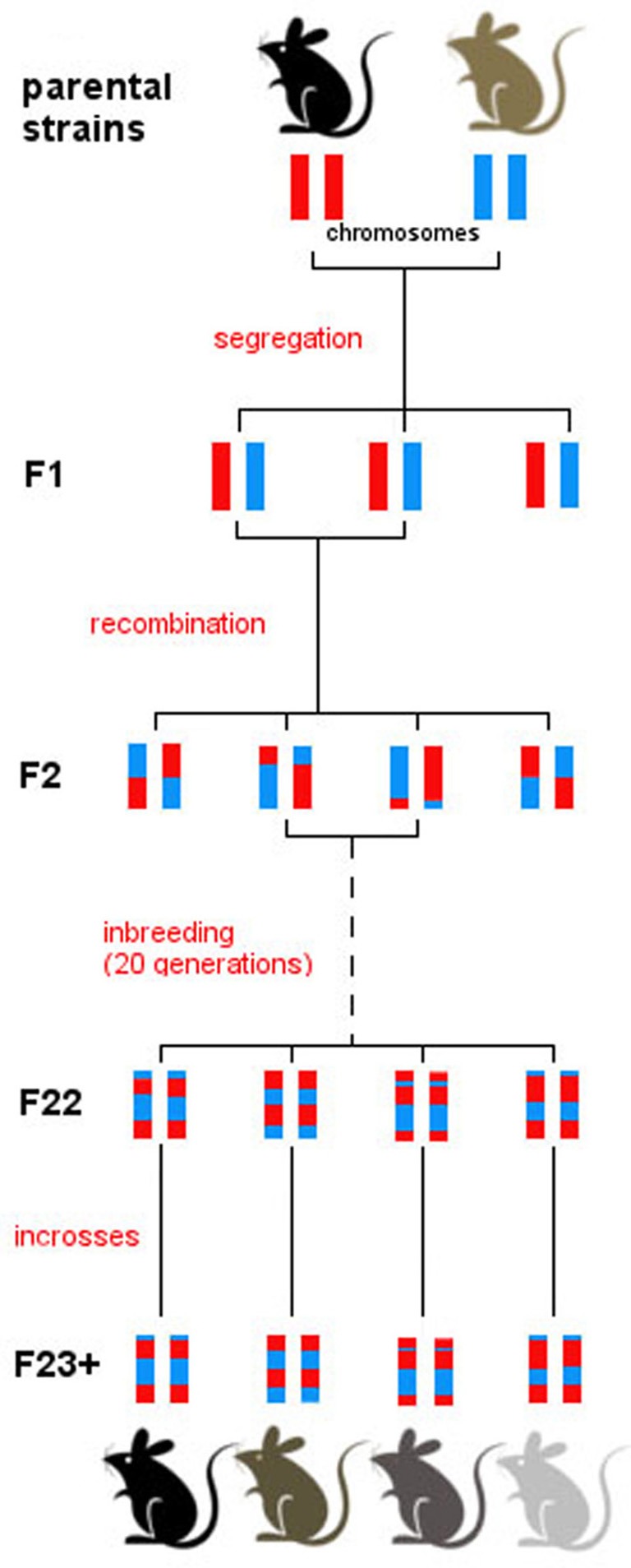
**Derivation of the BXD set.** All BXD lines are derived from two parental strains, namely C57Bl/6J and DBA/2J. Following a cross between the two, the F1 generation consists of genetically identical individuals that inherited one chromosome from each parental strain. Intercrosses were then carried out between F1 individuals, generating recombination in the F2. Patterns of recombination were frozen with 20 generations of sib-matings, which resulted in almost complete homozygosity in generation F23. From then on, breeding was continued by full-sib matings within every line, and individuals were monitored to ensure consistency in the genotype of each line over time. After generation F23, therefore, each line represents a unique mosaic of C57Bl/6J and DBA/2J alleles; there is extensive variation between line, and virtually no genetic variation between individuals of one line.

RI strains consist of many lines, each of which is defined by a fixed recombination pattern of exactly two possible alleles (Silver, 1995). For example, in the BXD strain, there are over 100 different lines. Animals in each of these lines have the same genotype but they vary across lines. RI animals are homozygous at all loci so they can be maintained indefinitely, but each line expresses a unique combination of parental alleles, exactly two possible alleles, as two parental strains were crossed. From a parental intercross families are derived and then continuously inbred within a family, thus “freezing” the unique recombination pattern of the resulting line (Figure 1). The BXD set was established from an intercross of the inbred mouse strains C57Bl/6J and DBA/2J, which differ in many phenotypic traits and are thus ideal to study behavioral traits (e.g., Boughter et al., 2007). Currently, the BXD set consists of 103 lines and was originally developed by Taylor in the late 1970's (lines BXD1–BXD43) (Chesler et al., 2005), with lines 43 upwards developed later by Lu Lu, Jeremy Peirce, Lee M. Silver, and Robert W. Williams.

The BXD set derives its high variability from the fact that its genome contains elements from *Mus musculus domesticus* (c. 92%), *M. m. musculus* (c. 7%), and *M. m. castaneus* (c. 1%) (Yang et al., 2007). 11% of the genome of BXD lines is identical by descent when all inbred strains are considered (Yang et al., 2007). Therefore, those non-polymorphic regions cannot be investigated in BXD. Further, statistical power is limited by the number of lines and is generally smaller compared with a unique intercross population between inbred lines (where each individual is genetically unique rather than each line in RI sets).

The development of the Collaborative Cross (CC), a RI panel developed from three wild-derived and five inbred strains (Churchill et al., 2004), promises much greater genetic diversity and higher resolution at gene level. The first studies from this system have recently been published (Threadgill and Churchill, 2012) and demonstrate the potential of the CC panel. In particular in combination with studies on BXD (high power but lower resolution), the CC panel allows the dissection of the genetic architecture of complex behavioral traits. Of course, genetic diversity is maximized in mice derived from recently caught wild mice (Guenet and Bonhomme, 2003).

**Figure 2:**
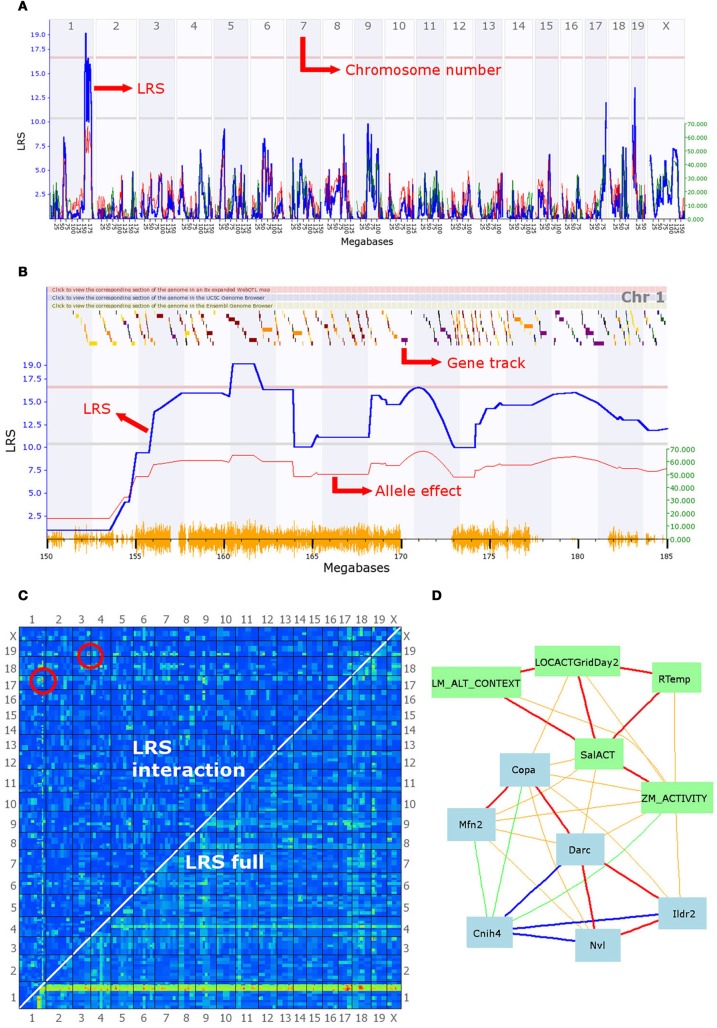
**A selection of the analytical tools available on GeneNetwork.** In this example, the focal trait (ID number 12361) is a behavioral phenotype (mouse activity in a maze) submitted by Cook et al. and defined as “Anxiety assay, baseline untreated control (BASE group), activity in closed quadrants using an elevated zero maze in 60–120 days-old males and females during 10 min [n beam breaks].” **(A)** Genome scan: the output of the interval mapping analysis. The program calculates the correlation between known SNPs and the phenotype values entered by the user. This generates a Likelihood Ratio Statistic (LRS) for each location on the genome, which is plotted as a blue line. A high LRS score suggests that genes associated with the trait are present at the location of significant SNP markers. Statistical significance thresholds are defined using a permutation test (*n* = 2000) and displayed on the graph as grey and red lines (suggestive and significant thresholds, respectively). This graph clearly shows a significant quantitative trait locus (QTL) on chromosome 1, possibly associated with additional linked loci, as well as two suggestive QTL on chromosomes 17 and 19. **(B)** The second graph shows a similar LRS plot, zoomed in on a section of chromosome 1 between 150 and 185 megabases. Multiple LRS peaks are visible, suggesting that multiple loci in the region may be affecting this phenotype in mice. The QTL found can be further investigated by examining correlation patterns and the gene expression databases (see description of panel D). The graph also presents an additional red line representing the magnitude of the effect of alleles, i.e., how much larger is the trait value on average in individuals with the B6 allele. This line is associated with the y-axis on the right-hand side. It indicates that mice possessing a B6 allele break the beam in the dark section of the maze 60 times more often than their counterparts, on average. The gene track at the top of the graph permits an initial exploration of the interval. Scrolling over a square reveals the gene at that location; squares are color-coded according to the number of SNPs existing between the B6 and DBA alleles as loci with greater polymorphism have a higher chance to be associated with differences in the phenotype. **(C)** Results of the epistasis analysis. The program analyzes the correlation between every possible pair of chromosome locations and the phenotype. Red and yellow colors indicate high LRS scores and therefore high chances of a gene associated with the trait at that location. The bottom right-hand half of the graph shows results for the combined single-gene and epistatic effects. Indeed, the red and yellow band at the bottom of the graph corresponds to the significant QTL highlighted in **(A)** and **(B)**, which achieves high combined LRS scores because of the strong single-gene effect of a few QTL. The top left-hand half of the graph shows the results for epistatic interactions only. Two pairs of epistatic loci with a significant association with the phenotype are circled in red. These suggest strong epistatic interactions between a gene on chromosome 19 and one on chromosome 3 and between a second pair of loci on chromosomes 17 and 1. Significance threshold are given in a table below the graph on the website, and can be compared with a table of LRS values. **(D)** A network graph summarizing the interactions between the focal phenotype, other behavioral phenotypes, and gene expression in the brain. The focal phenotype is labeled ZM_ACTIVITY. Other phenotypes are in green boxes, whereas gene expression data is in blue boxes. Red and orange lines represent positive correlations, blue and green ones represent negative correlations; the thickness of lines indicates the strength of the correlation. The traits in this graph were obtained by selecting the top 10 unique traits from the “best correlations” searches, but only traits fully connected in the graph are shown. The behavior phenotypes displayed are: LM_ALT_CONTEXT = “fear conditioning response,” LOCACTGridDay2 = “baseline locomotor activity using grid test,” Rtemp = “body temperature (rectal) of 13-week old males,” SalACT = “locomotion after [saline] injection.” Blue boxes contain gene symbols for which gene expression correlates with the focal phenotype. Hippocampus gene expression was used for *Cnih4* and *Nvl*, hypothalamus data for *Copa, Mfn2, Darc* and *Ildr2*. Firstly, this graph illustrates that behavior correlates in a number of contexts, including zero mazes, open field, and fear conditioning. Importantly, it also shows that some of the same genes correlate with many behavioral phenotypes. These two observations might be the first step towards an in-depth genetic analysis of behavioral syndromes. Moreover, some intriguing potential clues to the mechanisms involved are given. For instance, rectal temperature correlates with anxiety and locomotion, which in turn correlate with the expression of *Mfn2*, a gene involved in mitochondrial function and metabolism, and known to be associated with hypertension. Finally, it should be noted that *Copa, Darc*, and *Cnih4* are all located in the QTL interval on chromosome 1, and the correlation between their expression and the focal anxiety phenotype makes them good candidate genes for the behavior measure here.

The BXD set is characterized by 4–5 million segregating single nucleotide polymorphisms (SNPs), 500,000 insertions and deletions (indels), and 55,000 copy number variants (CNVs, 1–100 kb; Sachidanandam et al., 2001). Importantly, because recombination patterns are fixed in each line, data collected in separate experiments can be analyzed in conjunction since the genetic variation in the lines remains identical. This allows correlational analyses between phenotypes and may identify biological pathways and pleiotropic gene effects. Further, no genotyping is required to identify candidate genes: an established linkage map with currently ~3800 SNP markers is used for quantitative trait loci (QTL) mapping, the results of which highlight areas of the genome associated with any given trait. Those areas can be examined in detail down to the nucleotide level, thanks to the fact that over the past two years the DNA sequences of both underlying mouse strains (C57BL/6J, henceforth B6 and DBA/2J, henceforth DBA) have been established (Wang et al., 2010). Moreover, gene expression data for many cell and tissue types have been generated in a variety of conditions and are accessible on GeneNetwork. Further, an extensive online tool for statistical analysis is now available (GeneNetwork) that integrates phenotypes, gene expression and DNA data to allow detailed investigations (Figure 2).

## Genetic basis of traits: candidate genes and constraints

Understanding the genetic basis of phenotypic variation is crucial to a number of questions in behavioral ecology and often lies at the heart of the question why an animal behaves in a certain way and whether a behavior may be adaptive, or why not (Fitzpatrick et al., 2005). Can we identify candidate genes underlying behavior? How much does an animal's behavior depend on ecological conditions and is there genetic variation for a trait or strategy (i.e., it may respond to selection and evolve)? Are genes that underlie a specific behavior also causal to other phenotypes and are these possibly functionally related (Box 1)? A first step to answering such questions is the identification of locations in the genome that contain sequence variants (QTL) modifying the phenotype in question. QTL analysis examines the association between marker genotypes and phenotypes using, in this case, interval mapping (Lander and Botstein, 1989). Such analyses identify regions in the genome where alleles responsible for altering a particular phenotype may be located. Identifying these QTL also provides a degree of insight into what parts of the genome change when populations evolve (Grozinger, 2010). This commonly used approach has identified loci for a variety of behaviors, including anxiety in mice (Henderson et al., 2004; Sokoloff et al., 2011), foraging in bees (Rüppell et al., 2004) and mating calls in crickets (Ellison et al., 2011).

When running a QTL analysis, GeneNetwork generates an allele effect plot (in addition to the standard QTL plot), quantifying the effects of alleles at all possible locations on the phenotype. Using this tool, a QTL analysis may also shed light on whether differences in phenotype are due to one or two large effect genes or many loci of small effect (Stapley et al., 2010). A model constructed by Malcom (2011) highlights the importance of considering the genetic architecture when attempting to predict evolutionary trajectories by suggesting that a trait controlled by a small gene network will adapt more rapidly but reach a less than optimal endpoint, whereas a trait controlled by a large gene network will evolve more slowly but more accurately. In the BXD RI set the list of potential candidate genes with the region defined by a QTL can be further narrowed down by comparing the DNA sequences of the two founding strains DBA and C57 (Wang et al., 2010) searching for genes that show a functional polymorphism.

## Gene interactions

An element contributing to the complexity of the genotype–phenotype relationship is epistasis, where multiple genes interact to affect a phenotype, i.e., the effects of a genotype at one locus depend on the genotypes at other loci. Evolutionarily, epistasis is important because it can contribute to the additive genetic variance, which determines the response to selection (Wolf et al., 2000). Epistasis has been studied extensively using genome scans in the context of human disease, where modifier genes have long been known to alter or mask the effect of disease-related mutations (Nadeau and Dudley, 2011). In behavioral ecology, understanding epistasis patterns may raise new questions on the robustness of individuals in the face of deleterious mutations, opening up the possibility that selection may be less able to remove deleterious mutations from some populations due to the masking effects of modifier genes. Indeed, Weinreich et al. (2005) have demonstrated that functional epistasis (*sensu* Hansen and Wagner, 2001), where the effect of an allele depends on the genetic background at other loci, can alter evolutionary trajectories. Thus, if we wish to understand and predict evolutionary trajectories (for instance to model the effect of anthropogenic change on natural populations: Hellmann and Pineda-Krch, 2007; Yurk and Powell, 2009), investigations of epistasis in RI lines may provide insight into the genetic architecture of behavioral traits.

Behavioral ecologists often test predictions in animal systems derived from optimality models where simplifying assumptions are made about the key parameters the animals seeks to optimize. One of the key reasons for deviations from predictions may be genetic constraints imposed by genetic linkage to genes with antagonistic (fitness) effects (e.g., Gratten et al., 2008). In BXD, we can investigate positive or negative gene interaction using epistasis analyses but also by exploring correlations with other phenotypes that were collected in other studies. The direction of phenotypic correlation between traits may be an indicator of positive or negative genetic linkage and genetic analyses can then be focused on loci underlying the correlated traits or can be examined in more detail with the network graph and partial correlation tool (Figure 2D; for more information, see the online tutorials on http://webqtl.org/tutorial/ppt/index.html). If a set of traits has been identified to be part of the same pathway (e.g., high correlation and biologically plausible), pleiotropy can be investigated by plotting the set of traits onto a multiple QTL map, which superimposes the Likelihood Ratio Statistic (LRS) plot for all phenotypes selected. Because in a given study it will be impossible to measure many conceivably related phenotypes, RI sets offer the enormous advantage to draw on both gene expression and phenotypic data collected in different studies.

It has long been established that effects of genetic variation on phenotypic variation can depend on environmental conditions (e.g., Bradshaw, 1965; West-Eberhard, 1989), but only with genome-wide scans is it possible to identify novel genes whose effects on phenotypes depend on environmental conditions (e.g., Thomas, 2010). In behavioral ecology, it is likely, and somewhat unsurprising, that for example life history strategies depend on ecological conditions (Werner and Gilliam, 1984; Ludwig and Rowe, 1990; Ghalambor et al., 2010). However, to what degree a specific behavior may depend on environmental conditions, which genes (and thus traits) might be susceptible to quantifiable environmental modification (ranging from physical to social environment, e.g., diet or number of siblings) can be explored in detail in RI systems as it allows clear manipulation of environmental conditions on defined genetic backgrounds. For example, one can compare behavioral phenotypes measured across multiple RI lines under two different environmental conditions (e.g., diet, social environment) to identify genes that modify behavior in one environment but not the other.

## Conclusion

In systems biology the concept of gene networks has come to the forefront, with the proposal that many aspects of development, physiology, and behavior are controlled in a modular fashion (Grozinger, 2010; Nadeau and Dudley, 2011). Modules, or sets of genes acting in concert to generate individual aspects of biology such as metabolism, behavior etc., are thought to arise by necessity in organisms, causing constraints such as behavioral syndromes in animals (Sih et al., 2004). Large phenotypic changes such as the ones involved in speciation are thought to often arise from a change in the way modules interact with each other, rather than changes within the modules themselves (Grozinger, 2010). The systems genetics approach proposed in this perspective is ideal for testing the hypothesis of modularity. In particular, tools have been specifically designed to map gene networks and estimate their strength (Figure 2D); results from such analyses will begin to elucidate the relationships within and between modules.

The key to answering functional, mechanistic, and ontogentic questions about behavior relies on using a system that allows testing predictions using experimental manipulations, and for which detailed data from genetics to gene expression, physiology through to complex behavioral phenotypes can be obtained. Large RI panels such as BXD make this possible and are thus an ideal system to experimentally investigate key concepts in behavioral ecology. Moreover, we may be able to gain a comprehensive understanding of the genetic and environmental causes of phenotypic diversity among and within species.

### Conflict of interest statement

The authors declare that the research was conducted in the absence of any commercial or financial relationships that could be construed as a potential conflict of interest.
